# Mass azithromycin distribution for reducing childhood mortality in sub-Saharan Africa

**DOI:** 10.1056/NEJMoa1715474

**Published:** 2018-04-26

**Authors:** Jeremy D Keenan, Robin L Bailey, Sheila K West, Ahmed M Arzika, John Hart, Jerusha Weaver, Khumbo Kalua, Zakayo Mrango, Kathryn J Ray, Catherine Cook, Elodie Lebas, Kieran S O'Brien, Paul M Emerson, Travis C Porco, Thomas M Leitman

**Affiliations:** 1 Francis I Proctor Foundation, UCSF; 2 Department of Ophthalmology, UCSF; 3 London School of Hygiene & Tropical Medicine; 4 The Dana Center, Johns Hopkins University School of Medicine; 5 The Carter Center, Niger; 6 Blantyre Institute for Community Outreach, Blantyre, Malawi; 7 College of Medicine, University of Malawi, Blantyre, Malawi; 8 National Institute for Medical Research, Tanzania; 9 The International Trachoma Initiative; 10 Emory University; 11 Department of Epidemiology and Biostatistics, UCSF; 12 Institute for Global Health Sciences, UCSF

## Abstract

**Background.:**

Interventions to reduce under-5 mortality can either target the vulnerable or include all children regardless of state of health. Here, we assess whether mass distribution of a broad-spectrum antibiotic to pre-school children reduces mortality in sub-Saharan Africa.

**Methods.:**

MORDOR was a large simple trial that randomized communities in Malawi, Niger, and Tanzania to 4 biannual mass distributions of either oral azithromycin or placebo. Children aged 1-59 months were enumerated and offered treatment. Vital status was assessed at the subsequent biannual census. The primary outcome was aggregate all-cause mortality, with country-specific rates as pre-specified subgroup analyses.

**Results.:**

In total, 1533 communities were randomized, 190,238 children censused at baseline, and 323,302 person-years monitored. Mean antibiotic coverage over the 4 biannual distributions was 90.4% (SD 10.4%) of the censused population. The overall annual mortality rate in placebo- treated communities was 16.5 per 1000 person-years (9.6 per 1000 person-years in Malawi, 27.5 in Niger, and 5.5 in Tanzania). Antibiotic-treated communities had an estimated 13.5% lower mortality overall (95% CI 6.7%—19.8%, *P*<0.001). Mortality was 5.7% lower in Malawi (CI - 9.7%—18.9%, *P*=0.45), 18.1% lower in Niger (CI 10.0%—25.5%, *P*<0.001), and 3.4% lower in Tanzania (CI -21.2%—23.0%, *P*=0.77). The greatest reduction was observed in 1-5 month-old children (24.9% lower, CI 10.6%—37.0%, *P*=0.001).

**Conclusions.:**

Mass azithromycin distribution to post-neonatal, pre-school children may reduce childhood mortality in sub-Saharan Africa, particularly in high mortality areas such as Niger. Any implementation would need to consider selection for antibiotic resistance.

## Introduction

Trachoma control programs have distributed more than 600 million doses of oral azithromycin in an effort to eliminate the ocular strains of chlamydia that cause the disease.^1^,^2^ Azithromycin has been effective against trachoma, although distributions have caused gastrointestinal side effects and selected for macrolide-resistant strains of *Streptococcus pneumoniae* and *Escherichia coli*.^3^-^8^ Investigators have also noted possible benefits against a number of infectious diseases including malaria, diarrhea, and pneumonia.^9^-^14^ A case-control study and community-randomized trial in a trachoma-endemic area of Ethiopia suggested that mass azithromycin may even reduce childhood mortality.^15^,^16^ Experts believed a mortality benefit possible, although likely smaller in magnitude than found in these studies.^17^

Here, we tested the hypothesis that biannual mass distributions of oral azithromycin can reduce mortality in children aged 1-59 months. The study was performed in 3 geographically distinct areas: Malawi in Southern Africa, Niger in West Africa, and Tanzania in East Africa. Azithromycin affects transmissible diseases, so treating one individual might influence others in the same community. Thus randomization and intervention were at the community level, and inferences of efficacy were made at the community level. As mortality is a relatively rare event even in these settings, a large study population was required. Hence we adopted a large simple trial paradigm with a straightforward intervention and primary outcome.^18^

## Methods

### Eligibility

MORDOR (*Macrolides Oraux pour Réduire les Décès avec un Oeil sur la Résistance*) was a community-randomized trial conducted in the Malawian district of Mangochi, the Nigerien districts of Boboye and Loga, and the Tanzanian districts of Kilosa and Gairo. The randomization unit was a health surveillance assistant area in Malawi, a *grappe* in Niger, and a hamlet in Tanzania. Communities with a population between 200 and 2000 inhabitants on the most recent census were eligible for enrollment (Supplementary Appendix). Enrollment was based on census information available prior to the study. Communities remained in the study even if the population drifted out of this range. All children aged 1-59 months (truncated to month) weighing at least 3,800 grams were eligible for treatment.

### Randomization and masking

Lists of communities from the most recent pre-trial census were submitted to the UCSF data coordinating center. For each country, communities were randomly assigned in equal proportions to 1 of 10 letters, with 5 letters coded for azithromycin and 5 for placebo (Statistical Analysis Plan, SAP). Randomization was generated in R (R Foundation for Statistical Computing, Vienna, Austria) using the *sample* command (TCP), with knowledge of the link between treatment letter and arm assignment limited (TCP, KJR, and personnel necessary for labeling and packaging). Centralized randomization and simultaneous assignment of communities facilitated complete allocation concealment. Participants, observers, investigators, and data-cleaning team members were masked to treatment arm. The placebo contained the vehicle of the oral azithromycin suspension and was identical in appearance with identical bottles and labels.

### Census

A house-to-house census was performed during 5 prescribed 6-month periods, allowing a 2-month grace period for the initial census. At the initial census, all households in the community were entered into a custom-built mobile application (Conexus Inc., Los Gatos, CA), with the head of household name and GPS coordinates used to facilitate locating the household at the subsequent census. All children in the household aged 1-59 months were enumerated. Pregnant women and children under 1 month were also documented in anticipation of the following census. At follow-up censuses, the vital status (alive, dead, or unknown) and residence (living in community, moved outside community, or unknown) were recorded for children present in census records. New 0-59 month-old children and pregnant women were also entered. Communities were censused in the same general order throughout the study. Data were uploaded to the Salesforce Cloud Database Service (Salesforce.com, San Francisco, CA). Data cleaning was performed using Salesforce.com, Stata (Statacorp, College Station, TX), and R. 

### Intervention

Each child aged 1-59 months at the census was offered a single, directly observed dose of oral azithromycin or placebo (both provided by Pfizer, Inc., New York, NY). A volume of suspension corresponding to at least 20 mg/kg was given by height-stick approximation according to the country’s trachoma program guidelines, or by weight for those unable to stand. Children known to be allergic to macrolides were not treated. Treatments were administered at the census and during additional visits in an attempt to achieve at least 80% coverage. Administration of study medication was documented for each child in the mobile application, and community coverage was calculated relative to the census. Guardians were instructed to contact a village representative for any adverse events noted after taking the study medication. This individual reported to the site study coordinator, who in turn reported to the Data Coordinating Center at UCSF. 

### Primary outcome

The pre-specified primary outcome was the community-level, aggregate, 3- country mortality rate determined by biannual census. Each inter-census period was treated separately, with a mortality event counted only when a child was recorded as being alive and living in the household at the initial census, and recorded as having died while residing in the community at the subsequent census. By design, no attempt was made to track down a child’s status after movement outside the community. Person-time at risk was calculated as days between consecutive censuses, with children who moved, died, or had an unknown follow-up status contributing one half the inter-census period. All children documented as alive and living in the household at the initial census of each inter-census period were included in the analysis. No changes to trial outcomes were made after the trial had commenced.

### Subgroup analyses

Mortality rates were assessed by country site and age group. An abbreviated version of the 2007 World Health Organization verbal autopsy questionnaire for children aged 4 weeks to 14 years was used to collect data for verbal autopsies.^19^ Causes of death were assigned using an algorithm based on a published verbal autopsy hierarchy.^20^

### Sample size and statistical analysis plan

We estimated that inclusion of 620 communities per country would provide at least 80% power to detect an overall reduction in all-cause mortality of 10%. Specifically, we assumed mortality rates of between 14 and 20 per 1000 child-years, average community sizes of 600 to 799 people (16.7% to 19.0% of which were children aged 1- 59 months), coefficients of variation of between 0.40 and 0.51, and loss to follow-up of 10% (SAP).

The pre-specified primary analysis was negative binomial regression of the number of deaths per community, with treatment arm and country as predictors and total person-time at risk as an offset. All 3 country sites contributed to the primary outcome. Hypothesis testing was 2-sided, allowing a total alpha of 0.05 for the interim and final analyses. A *P*-value was determined by Monte Carlo permutation testing (10,000 replications). An interim efficacy analysis after the 12- month census was designed to spend 0.001 of the total alpha, reserving an alpha of 0.049 for the primary 24-month analysis. Community-level clustering was taken into account by the dispersion parameter in the negative binomial regression. Pre-specified subgroup analyses included negative binomial regression of community-level mortality rates by country, age group, and inter-census period (SAP). A sample of 250 verbal autopsies from each site were compared using the chi-squared statistic, with clustering taken into account by permutation at the community level. All statistical analyses were conducted in R. 

### Ethics

Approval for the study was obtained from the ethical committees of the College of Medicine, University of Malawi, Blantyre, the Niger Ministry of Health, and the Tanzanian National Institute for Medical Research, as well as London School of Hygiene & Tropical Medicine, UCSF Committee for Human Research, Emory University, and Johns Hopkins University School of Medicine. Informed consent was obtained from the local Ministries of Health, village leaders, and guardians of children. No incentives were offered for participation, although all children in the Niger site were offered azithromycin at the conclusion of the study, and those in Malawi entered into the country’s trachoma program. The study was undertaken in accordance with the Declaration of Helsinki.

A Data and Safety Monitoring Committee provided oversight. Members of the investigator steering committee (Supplementary Appendix) designed the trial, vouch for its adherence to the protocol, and attest to the accuracy and completeness of the data and analyses as specified in the protocol and SAP. The corresponding author wrote the initial draft, and all coauthors reviewed the manuscript and agreed to publication.

## Results

### Participant flow

**Figure 1.**
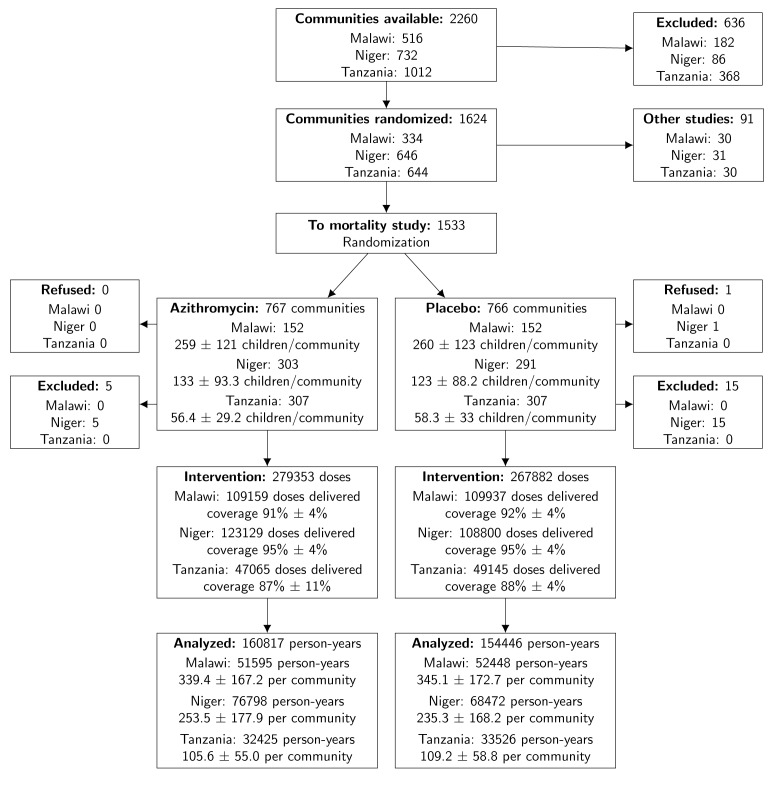
Enrollment, Randomization, and Treatment

As displayed in Figure 1, 1624 of 2260 communities in the chosen districts were eligible and randomized to either this study (1533) or parallel studies (91). In Niger, 1 community refused to participate, and 20 were excluded due to being misspelled duplicates, nonexistent at the time of census, or indistinguishable from larger neighboring communities. No randomization units were lost to follow-up after the initial census.

Census periods started in December 2014, August 2015, February 2016, August 2016, and February 2017. Data collection was completed by July 2017 and the database closed for primary analysis on October 15, 2017. Baseline characteristics of the communities are displayed in Table 1. 323,302 person-years were monitored over the 5 census visits, including 111,559 person-years in Malawi, 145,597 person-years in Niger, and 66,146 person-years in Tanzania. To validate the census data collection, a subset of households was censused by an independent field team later during the census period. The majority of recensused children had been enumerated on the first census: 95% (257/271) for Malawi, 92% (286/310) for Niger, and 95% (4544/4791) for Tanzania. Coverage of the targeted population of children was 90.4% (standard deviation 10.1%) in the placebo arm and 90.3% (±10.6%) in the azithromycin arm: in Malawi, antibiotic coverage was 91.5% (±6.4%) in the placebo arm and 91.5% (±6.1%) in the azithromycin arm, in Niger 94.5% (±6.7%) in the placebo arm and 94.5% (±6.0%) in the azithromycin arm, and in Tanzania 86.1% (±12.3%) in the placebo arm and 85.5% (±13.6%) in the azithromycin arm (Supplementary Appendix).

**Table 1.** Baseline characteristics in the azithromycin and placebo treated arms

	All Countries		Malawi		Niger		Tanzania	
	Azithromycin	Placebo	Azithromycin	Placebo	Azithromycin	Placebo	Azithromycin	Placebo
Communities	762	750	152	152	303	291	307	307
Children	97,047	93,191	39,386	39,534	40,345	35,747	17,316	17,910
Children/community (mean ± sd)	171 ± 126	169 ± 128	259 ± 121	260 ± 123	133 ± 93	123 ± 88	56 ± 29	58 ± 33
Male	50.7%	50.6%	50.2%	50.0%	51.2%	51.4%	50.5%	50.6%
Age								
1-5 months	7.4%	7.4%	7.0%	6.9%	6.8%	6.9%	9.4%	9.2%
6-11 months	13.2%	13.2%	12.3%	12.3%	13.5%	13.6%	14.5%	14.5%
12-23 months	19.1%	19.2%	20.5%	20.2%	17.0%	16.8%	21.0%	21.8%
23-59 months	60.4%	60.2%	60.2%	60.6%	62.8%	62.7%	55.1%	54.5%

### Primary results

The annual mortality rate for eligible children in the placebo-treated communities in the 3 countries combined was 16.5 per 1000 person-years (9.6 per 1000 person- years in Malawi, 27.5 per 1000 person-years in Niger, and 5.5 per 1000 person-years in Tanzania). A 12-month interim assessment for efficacy did not trigger the pre-specified early stopping rule (set at *P*<0.001). Over all 4 inter-census periods, community-level, intention-to- treat analysis revealed that the azithromycin-treated arm had 13.5% lower mortality overall (95% CI 6.7%—19.8%, *P*<0.001). The proportion of children whose census status was recorded as moved or unknown was not significantly different between the two arms (*P*=0.71 and *P*=0.36 respectively).

### Subgroup results

**Figure 2.**
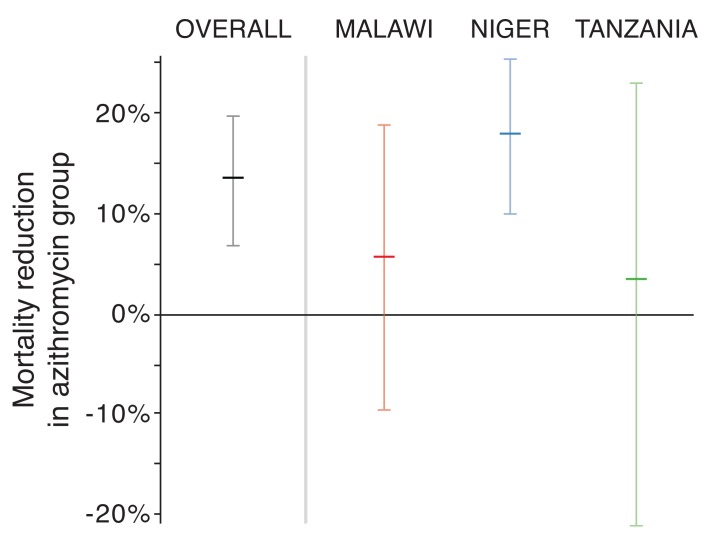
Efficacy of azithromycin overall and by country

**Figure 3.**
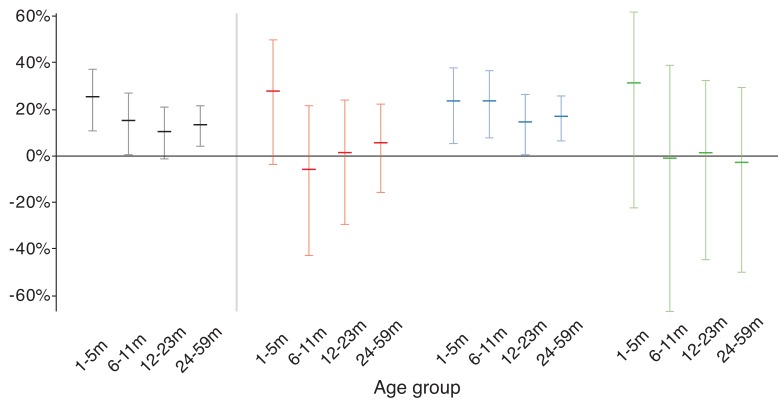
Efficacy of azithromycin by age

**Figure 4.**
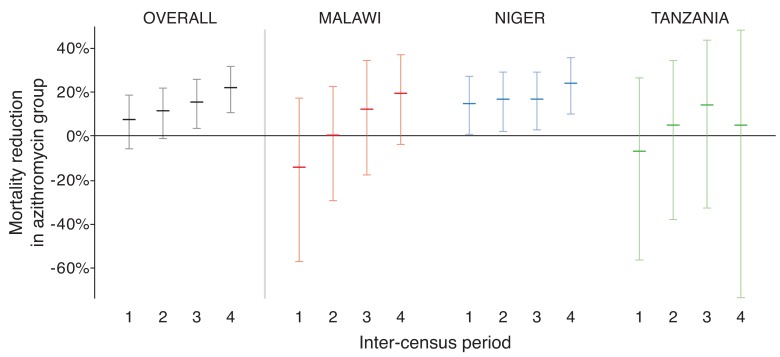
Efficacy of azithromycin over time

Mortality rates in the azithromycin-treated arm were 5.7% lower in Malawi (- 9.7%—18.9%, *P*=0.45), 18.1% lower in Niger (CI 10.0%—25.5%, *P*<0.001), and 3.4% lower in Tanzania (CI -21.2%—23.0%, *P*=0.77; Figure 2). Children in the 1-5 month old age group had the highest overall mortality and the largest observed reduction in mortality with azithromycin (24.9% reduction, 95% CI 10.6%—37.0%, *P*=0.001, Figure 3). The estimated efficacy of azithromycin increased with each inter-census period, going from 7.3% (CI -5.9% to 18.8%, *P*=0. 26) in the first period to 22.0% (CI 10.6% to 31.9%, *P*<0.001) in the last period (Figure 4). Efficacy was not significantly different by country site (*P*=0.17), age group (*P* =0.20), treatment period (*P* =0.09), or treatment coverage (*P* =0.34).

### Serious adverse events and overall causes of death

Not including the primary outcome of mortality, 20 hospitalizations or life-threatening illnesses occurred: 1 from Malawi, 3 from Niger, and 16 from Tanzania. 11 events were in the treated arm and 9 in the untreated arm. Medical review was unable to declare that any serious adverse event was probably caused by azithromycin. A random sample of 250 verbal autopsies from each of the 3 sites estimated that 41% of deaths were due to malaria, 18% diarrhea or dysentery, and 12% pneumonia (Supplementary Appendix). Cause of death was significantly different between the country sites (*P*<0.001), with relatively more deaths attributed to malaria in Niger and pneumonia in Tanzania.

## Discussion

Biannual distribution of oral azithromycin to post-neonatal preschool children significantly reduced all-cause mortality by approximately 14%. A majority of the deaths and of the observed effect was seen in Niger, which had an 18% reduction. In subgroup analysis, only the Niger site revealed a statistically significant reduction of mortality. The overall 14% effect is less than that seen in a previous case-control study and community-randomized trial in Ethiopia, but is in line with the 18% effect that a group of experts had anticipated when polled before the study.^15^-^17^

Azithromycin was most effective in children aged 1-5 months, preventing 1 in 4 deaths. The United States Food and Drug Administration has not approved azithromycin for children in that age group, and the World Health Organization does not currently recommend including them in trachoma distributions.^21^ However, the Centers for Disease Control does recommend oral azithromycin for all ages for treatment and prophylaxis of pertussis.^22^ Any mass distribution below 1 month of age would need to consider the risk of inducing infantile hypertophic pyloric stenosis (IHPS).^23^-^25^

This study did not investigate the mechanism by which azithromycin reduced mortality. Before the trial, experts thought a protective effect would most likely be due to reductions in respiratory infections, diarrhea, and malaria, in that order.^17^ Such a hypothesis seems reasonable, given azithromycin’s activity against bacterial pathogens of the lung and gastrointestinal tract, and the plasmodial apicoplast. Further study will be necessary to identify how azithromycin prevents mortality. Investigation is already underway. Smaller parallel trials with detailed microbiological and anthropometric assessments were conducted at each study site. Inference from these smaller trials will be directly applicable to the mortality result, because they drew communities at random from the same pool as the parent trial. Azithromycin has been linked to cardiac death in adults, although results are mixed and may not be relevant to children in this setting.^26^-^29^ This community-based trial, and even the more detailed parallel studies, did not have the capacity to monitor QT intervals as would be possible in a hospital-based setting.^28^

Non-specific antibiotic use is discouraged due to concern over antibiotic resistance. Repeated mass azithromycin distributions for trachoma select for macrolide resistance in nasopharyngeal *S. pneumoniae* and rectal *E. coli.*^6^,^7^,^30^,^31^ Resistance emerging during mass azithromycin distributions could curb or even reverse any potential mortality benefit. We did not observe such a waning effect—in fact, the observed effect increased from 7% to 22% over the 4 biannual inter-census periods. Nonetheless, longer follow-up is warranted to determine whether the mortality effect observed in the present trial changes with subsequent rounds of treatment.

The study had several limitations. As a large simple trial, little information was collected on each child and community. Deaths were determined by consecutive censuses. Children who were born and died between censuses did not contribute to either death count or person-time at risk for the primary outcome. Secondary analyses may reveal whether these children were better off being in a treated community even if they themselves were not born in time for treatment. No effort was made to follow children after they had moved. Death rates may have differed in children who moved or had an unknown census status. As distributions were offered only biannually, a child’s first treatment might not be until 7 months of age. Supplementary treatments given to infants during a scheduled vaccination visit to a health clinic could potentially add benefit. While mortality is seasonal, communities were treated in a rolling fashion over each 6-month time period for logistical reasons. Secondary analyses may reveal whether the drug was particularly effective in certain seasons. Lastly, the study was performed in 3 geographically diverse sites in Africa, but the results may not be generalizable outside of these districts. In fact, subgroup analyses only confirmed a statistically significant reduction in 1 of the 3 sites.

Two doses of oral azithromycin per year significantly reduced childhood mortality across 3 geographically diverse settings in sub-Saharan Africa. The largest effect was found in Niger, which has one of the highest child mortality rates in the world. Identifying specific mechanisms for mortality reduction will require further investigation. Any policy recommending mass distribution of oral azithromycin for childhood mortality would need to consider not only cost, but also the potential for antibiotic resistance.^32^

## Supplementary Material

Supplementary Appendix
