# Supplementary material for: Mass azithromycin distribution for reducing childhood mortality in sub-Saharan Africa
Source: N Engl J Med. Author manuscript; Available in PMC 2018 Apr 26. (PMC5849140; doi:10.1056/NEJMoa1715474)
Supplement: Supplementary Appendix [file NEJMoa1715474_Lietman_SupplementalAppendix.pdf]

APPENDIX

Table of Contents

Research Team……………………………………………………………………………………………... 2
Acknowledgements………………………………………………………………………………………….. 2
Table S1: Aggregate treatment coverage by arm and period…………….…………………………………… 3
Table S2: Treatment coverage by country, arm, and period…………………….……………………………… 3
Table S3. Census results………………………………………………………………………………………… 4
Table S4: Verbal Autopsy…………………………………………………………………...……………… 4
Table S5. Deaths and person-time-at-risk by age group…………………………………………………….…. 5
Table S6. Reduction of mortality with oral azithromycin by age group…………………………………………… 5
Table S7. Deaths and person-time-at-risk by inter-census period……………………………………………………6
Table S8. Reduction of mortality with oral azithromycin by inter-census period………………………………… 6

Research Team

In addition to the authors, the following investigators participated in MORDOR: *University of California, San Francisco, San Francisco, CA, USA* – Thuy Doan, Catherine E Oldenburg, Sun Y Cotter, Nicole E Stoller, Benjamin Vanderschelden, Dionna M Fry, Philip J Rosenthal, George W Rutherford, Zhaoxia Zhou, Lina Zhong; *London School of Hygiene and Tropical Medicine, London, UK* – David CW Mabey, Sarah E Burr, Anthony W Solomon; *Johns Hopkins University, Baltimore, MD, USA* – Kurt Dreger, Beatriz Munoz, Christian L Coles, Alain B Labrique, Alfred Sommer, Hemjot Kaur, Evan M Bloch; *Blantyre Institute for Community Ophthalmology, Blantyre, Malawi* – Alvin Chisambi, Zachariah Kamwendo; *University of Malawi College of Medicine, Blantyre, Malawi* – Ken Maleta; *The Carter Center, Atlanta, GA, USA* – E Kelly Callahan, Aisha E Stewart; *The Carter Center Niger, Niamey, Niger* – Salissou Kane; *Programme National de Santé Oculaire, Niamey, Niger* – Amza Abdou, Boubacar Kadri, Nassirou Beido; *Muhimbili University of Health and Allied Sciences, Dar es Salaam, Tanzania* – Mabula Kasubi; *National Institute for Medical Research, Dar es Salaam, Tanzania* – Leonard Mboera.

The steering committee for the trial consisted of the following investigators: Robin L Bailey, Jeremy D Keenan, Thomas M Lietman, Travis C Porco, and Sheila K West.

Acknowledgements

We thank the program officers from the trial's sponsor: *Bill & Melinda Gates Foundation,Seattle, WA, USA* – Rasa Izadnegahdar, Julie Jacobson, Thomas Kanyok, Erin Shutes. We also thank the members of the Data and Safety Monitoring Committee: *University of Washington, Seattle, WA, USA* – Judd L Walson; *Liverpool School of Tropical Medicine, Liverpool, UK* – Allen W Hightower; *Loyola University, Chicago, IL, USA* – Emily E Anderson, *Berhan Public Health & Eye Care Consultancy, Addis Ababa, Ethiopia* – Wondu Alemayehu; *Tulane University, New Orleans, LA, USA* – Latha Rajan.

Table S1: Aggregate treatment coverage by arm and period for all three countries

Inter-census period	Mean (± SD)	
	Azithromycin	Placebo
1	92.7% (±10.9%)	92.8% (±10.6%)
2	86.5% (±11.3%)	86.7% (±10.8%)
3	90.1% (±11.2%)	90.5% (±10.5%)
4	91.7% (±7.7%)	91.8% (±7.1%)
All	90.3% (±10.6%)	90.4% (±10.1%)

Coverage over all countries, all phases, and both arms was 90.4% (±10.4%).

Table S2: Treatment coverage by country, arm, and period

Inter-census period	Malawi Mean (± SD)		Niger Mean (± SD)		Tanzania Mean (± SD)	
	Azithromycin	Placebo	Azithromycin	Placebo	Azithromycin	Placebo
1	88.0% (±6.5%)	88.5% (±6.8%)	98.0% (±5.3%)	97.5% (±7.8%)	89.9% (±14.1%)	90.5% (±12.7%)
2	89.9% (±6.2%)	89.9% (±7.0%)	91.5% (±6.4%)	91.8% (±6.2%)	80.0% (±13.7%)	80.2% (±12.3%)
3	93.6% (±4.7%)	93.8% (±4.3%)	95.8% (±4.9%)	96.2% (±4.5%)	82.8% (±13.6%)	83.4% (±12.3%)
4	94.6% (±4.5%)	94.0% (±5.4%)	92.9% (±5.2%)	92.4% (±6.2%)	89.2% (±10.0%)	90.1% (±8.2%)
All	91.5% (±6.1%)	91.5% (±6.4%)	94.5% (±6.0%)	94.5% (±6.7%)	85.5% (±13.6%)	86.1% (±12.3%)

Table S3. Census results

Category	All Countries		Malawi		Niger		Tanzania	
	Azithromycin	Placebo	Azithromycin	Placebo	Azithromycin	Placebo	Azithromycin	Placebo
Census enrollments	**371592**	**358815**	**139150**	**141300**	**161257**	**143280**	**71185**	**74235**
Died	2404	2616	502	542	1727	1888	175	186
Moved	24415	23123	8351	7710	10084	8760	5980	6653
Unknown	18809	17587	11845	12160	6087	4508	877	919
Alive	325964	315489	118452	120888	143359	128124	64153	66477

S4: Verbal Autopsy

Cause	Malawi	Niger	Tanzania
Diarrhea/Possible Diarrhea	6/39	1/48	12/16
Dysentery/Possible Dysentery	1/1	1/2	4/2
Injury	7	5	13
Malaria/ Possible Malaria	48/55	84/38	65/19
Malnutrition	9	3	24
Measles	0	1	1
Meningitis	11	11	8
Other Infection	9	10	1
Pneumonia/Possible Pneumonia	8/18	8/8	27/20
Unspecified	38	30	38

Random sample of 250 deaths per country

P<0.001 (permutation p-value clustered by community)

Table S5. Deaths and person-time-at-risk by age group. (Totals can be affected by rounding.)

Age (mos)	Malawi Deaths / Person-years		Niger Deaths / Person-years		Tanzania Deaths / Person-years	
	Azithromycin	Placebo	Azithromycin	Placebo	Azithromycin	Placebo
1-5	57 / 3617	82 / 3739	202 / 5653	244 / 5148	21 / 2189	31 / 2229
6-11	102 / 5747	98 / 5912	286 / 7901	340 / 7143	41 / 3599	39 / 3595
12-23	131 / 11639	136 / 11897	491 / 15207	511 / 13573	54 / 7562	57 / 7924
24-59	212 / 34269	226 / 34738	748 / 48113	793 / 42860	59 / 19162	59 / 19886

Table S6 (for Figure 3, main text): Reduction of mortality with oral azithromycin by age group

Age (mos)	All Countries		Malawi		Niger		Tanzania	
	Estimate (95%CI)	*P*	Estimate (95%CI)	*P*	Estimate (95%CI)	*P*	Estimate (95%CI)	*P*
1-5	24.9% (10.6% to 37.0%)	0.001	27.6% (-4.1% to 49.7%)	0.08	23.1% (4.9% to 37.8%)	0.02	31.2% (-22.7% to 61.4%)	0.21
6-11	14.2% (0.0% to 26.3%)	0.05	-6.2% (-43.2% to 21.2%)	0.69	23.3% (7.4% to 36.3%)	0.01	-1.4% (-67.1% to 38.5%)	0.96
12-23	10.0% (-1.9% to 20.4%)	0.10	0.7% (-29.5% to 23.9%)	0.96	14.0% (0.1% to 26.0%)	0.048	0.9% (-45.0% to 32.3%)	0.96
24-59	12.6% (3.6% to 20.8%)	0.01	5.0% (-16.1% to 22.2%)	0.62	16.5% (6.1% to 25.8%)	0.003	-3.3% (-50.4% to 29.0%)	0.86

Mortality reduction was estimated from a negative binomial regression modeling community-specific deaths as a function of treatment arm, age group, and the treatment by age group interaction, with person-time at risk used as an offset. Positive numbers indicate a mortality reduction with azithromycin relative to placebo.

Table S7. Deaths and person-time-at-risk by inter-census period. (Totals can be affected by rounding.)

Inter-census period	Malawi Deaths / Person-years		Niger Deaths / Person-years		Tanzania Deaths / Person-years	
	Azithromycin	Placebo	Azithromycin	Placebo	Azithromycin	Placebo
1	118 / 12068	105 / 11998	531 / 23318	557 / 20549	64 / 9016	61 / 9311
2	159 / 15861	159 / 15935	382 / 17281	427 / 16015	53 / 8773	57 / 8948
3	97 / 12146	115 / 12678	439 / 18753	466 / 16744	38 / 7439	46 / 7767
4	128 / 15197	163 / 15676	375 / 17521	438 / 15415	20 / 7284	22 / 7608

Table S8 (for Figure 4, main text): Reduction of mortality with oral azithromycin by inter-census period

Inter-census period	All Countries		Malawi		Niger		Tanzania	
	Estimate (95% CI)	*P*	Estimate (95% CI)	*P*	Estimate (95% CI)	*P*	Estimate (95% CI)	*P*
1	7.3% (-5.9% to 18.8%)	0.26	-14.1% (-57.7% to 27.3%)	0.42	15.0% (0.6% to 27.3%)	0.04	-7.1% (-56.7% to 26.8%)	0.72
2	11.2% (-1.1% to 22.1%)	0.07	-0.0% (-29.5% to 22.8%)	>0.99	17.0% (2.1% to 29.5%)	0.03	5.1% (-38.4% to 34.9%)	0.79
3	15.7% (3.7% to 26.2%)	0.01	12.2% (-17.4% to 34.3%)	0.38	16.9% (2.6% to 29.2%)	0.02	13.8% (-32.8% to 44.1%)	0.50
4	22.0% (10.6% to 31.9%)	<0.001	19.3% (-4.1% to 37.4%)	0.10	24.1% (10.3% to 35.7%)	0.001	5.1% (-74.0% to 48.2%)	0.87

Mortality reduction was estimated from a negative binomial regression modeling community-specific deaths as a function of treatment arm, inter-census period, and the treatment by inter-census period interaction, with person-time at risk used as an offset. Positive numbers indicate a mortality reduction with azithromycin relative to placebo.
